# Nowcasting COVID-19 Statistics Reported with Delay: A Case-Study of Sweden and the UK

**DOI:** 10.3390/ijerph20043040

**Published:** 2023-02-09

**Authors:** Adam Altmejd, Joacim Rocklöv, Jonas Wallin

**Affiliations:** 1 Swedish Institute for Social Research, Stockholm University, 106 91 Stockholm, Sweden; 2 Department of Finance, Stockholm School of Economics, 113 83 Stockholm, Sweden; 3 Heidelberg Institute of Global Health (HIGH), Interdisciplinary Centre for Scientific Computing (IWR), Heidelberg University, 69117 Heidelberg, Germany; 4 Department of Public Health and Clinical Medicine, Umeå University, 901 87 Umeå, Sweden; 5 Department of Statistics, Lund University, 221 07 Lund, Sweden

**Keywords:** COVID-19, nowcasting, prediction

## Abstract

The COVID-19 pandemic has demonstrated the importance of unbiased, real-time statistics of trends in disease events in order to achieve an effective response. Because of reporting delays, real-time statistics frequently underestimate the total number of infections, hospitalizations and deaths. When studied by event date, such delays also risk creating an illusion of a downward trend. Here, we describe a statistical methodology for predicting true daily quantities and their uncertainty, estimated using historical reporting delays. The methodology takes into account the observed distribution pattern of the lag. It is derived from the “removal method”—a well-established estimation framework in the field of ecology.

## 1. Introduction

The coronavirus pandemic has profoundly affected societies all around the world. As countries were challenged to control and fight back, they were in need of timely, unbiased data to monitor trends and make fast and well-informed decisions [1]. One of the main areas of failure identified by the Lancet commission on COVID-19 was “the lack of timely, accurate, and systematic data on infections, deaths, viral variants, health system responses, and indirect health consequences” [2]. Official statistics are usually reported with a long delay after thorough verification, but in the midst of a deadly pandemic, real-time data are of critical importance for policymakers [3]. The latest data are often not finalized, but change as new information is reported. In fact, reporting delays mean that the most recent days have the least cases accounted for, producing a dangerous illusion of an always improving outlook.

Still, these unfinished statistics offer crucial information. If the pandemic is indeed slowing, we should not wait for the data to be finalized before using them. In Sweden, these statistics were reported with a warning sign, indicating that more cases would likely be reported in the future. In this paper, we argue that by explicitly taking the historical reporting delay into account, it is possible to produce more accurate statistics. When case counts and deaths are nowcasted to account for reporting delay, policymakers can use the latest numbers without being misled by reporting bias.

Predictions produced by a statistical model provide an additional feature that is perhaps even more important: they model the uncertainty about these unknown quantities, ensuring that all users of these data have the same view of the current state of the epidemic.

In this paper, we describe a statistical methodology for nowcasting epidemic statistics, such as hospitalizations or deaths, and the degrees of uncertainty surrounding these counts. The model is based on the daily reported event frequency and the observed distribution of reporting delays. The prediction model builds on a methodology developed in ecology, often referred to as the “removal method” [4]. We further show that this model is considerably more accurate and precise than the simpler strategy of simply including the average historical reporting delay.

To help motivate why such forecasting is needed, we now turn to the case of Sweden. The model is flexible by design, however, and in our Results section, we present data from both Sweden and the UK.

### Reporting of COVID-19 Statistics in Sweden

The publication of real-time pandemic statistics enables public health professionals and the public to follow the evolving patterns of the pandemic [5]. It is of specific interest to identify when the growth rate changes, since such events could indicate the need for a policy response. The Swedish Public Health Agency updates their public COVID-19 statistics daily [6]. By downloading these data each day, the authors of this paper have tracked the reporting delay since the very beginning of the pandemic. See the Supplementary Material for an archive of these reports.

At the peak of the pandemic, the Swedish Public Health Agency held daily press conferences where updates on the number of deaths, admissions to hospitals and intensive care, as well as case counts, were presented [7]. Unlike, e.g., the widely used trackers published by Our World in Data [8] and the Johns Hopkins Coronavirus Resource Center [9], where the statistic reported is “Daily new confirmed COVID-19 deaths”, the Swedish Public Health Agency reported deaths by event date. Each day, new numbers of confirmed deaths were reported and assigned to their actual date of death. This means that, when the daily statistics are reported by the Public Health Agency, not all events that have happened up to that day have yet been registered. Furthermore, the share of registered events is lowest for the latest dates. As a consequence, presenting the numbers by event date creates the illusion of a downward trend.

By instead presenting mortality statistics by the date they were reported, like Our World in Data, no such illusion is created. However, since such reports wrongly attribute deaths from many days back to the current date, any changes in growth rates will also show up with a delay. If the reporting delay changes for any reason, for example because of a weekend or a public holiday, this change will show up as a trend shift in such data.

**Figure 1:**
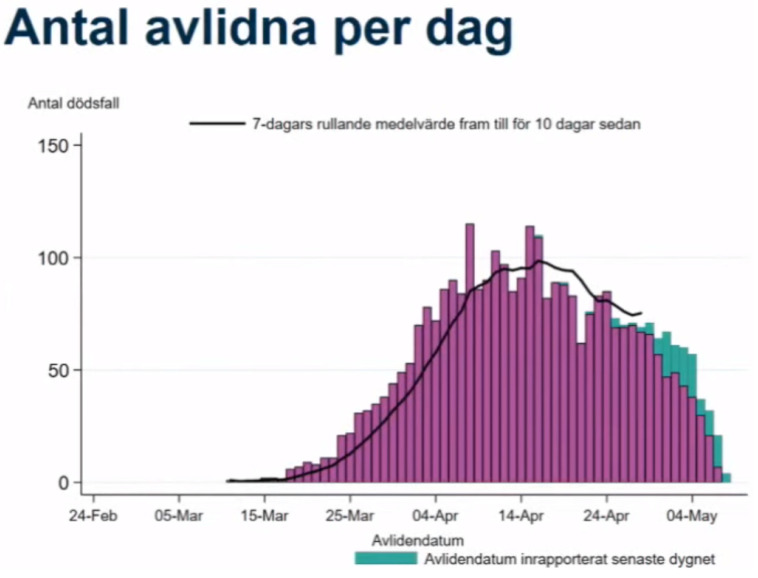
Screenshot from the daily press conference on 8 May by the Swedish Public Health Agency. The headline translates to “Number of deceased per day”. The bars show the number of deceased individuals who have been reported so far, assigned to their true date of death. The sections colored green show those who have been reported dead within the last 24 hours, while purple-colored bars represent earlier reported deaths. A 7-day rolling average of daily deaths is plotted as a black line. It stops on April 28, 10 days before the relevant date, because of the delay in reporting.

Reporting delays are different for different statistics. Deaths, which are in many ways the least biased measure of incidence, suffer from the longest reporting delays. The Swedish Public Health Agency tried to account for this in their press conference by reporting 7-day average trends 10 days prior to the latest date. However, in fact, deaths are often reported with a delay of more than 10 days, and the presentation of this information using a bar plot gives the false impression of a downward trend even when the cases are rising. Figure 1 displays a screenshot from the daily press conference on 8 May 2020. Once all deaths had been reported, 80 individuals had died on that date. The black line runs until 28 April. During this press conference, 69 individuals had been reported dead on 28 April—a number that would increase to 83 once reporting had finished more than a month later. In other words, while the Public Health Agency was aware of the reporting delay, they severely underestimated its magnitude. In fact, this might be the reason that the numbers of daily deaths have been underestimated by decision makers repeatedly. At the peak of the first wave, deaths were initially believed to level out at around 60 per day, but after all cases had been reported more than two weeks later, the actual number was close to 120 [10].

Reporting statistics by report date hides the illusory downward trend, but also throws away potentially useful information about when these newly reported events happened. Various institutional factors, such as weekends and work holidays, cause regular changes in reporting delay that are unrelated to the underlying trends in incidence. To rely on such measures is thus far from optimal. Instead, we propose a method to predict (nowcast) the actual number of daily deaths, making use of all the available information, including data about actual death date and the time it took for the death to be reported.

## 2. Materials and Methods

We propose to use the removal method, developed in animal management [4], to estimate the actual frequencies on a given day and their uncertainty. The method has a long history, dating back at least to the 1930s [11]. However, the first refined mathematical treatment of the method is credited to [12], and more modern derivatives exits today [13]. It is a commonly applied method today when analyzing age cohorts in fishery and wildlife management.

The removal method has three major advantages over simply reporting moving averages:

- It does not relay any previous trend in the data,
- It allows the generation of prediction intervals for the uncertainty of daily frequencies,
- These uncertainty estimates can be carried over to epidemiological models to increase realism.

A classic example where the method proposed to solve this problem has been used is in estimating statistics when trapping a closed population of animals [4]. Each day, the trapped animals are collected and kept. As long as there is no migration, the researcher will (on average) trap fewer animals each following day, given that a proportion of the total population is removed. This pattern of a declining number of trapped animals allows one to draw inference of the underlying population size, under the assumption of equal probability of catching animals. Our “animal population” is the true number of deaths (or other events) on a given day. As these numbers (for the specific day) are fixed, they can be clearly viewed as a closed population. Instead of traps, we have the new reports of COVID-19 deaths. Using the pattern of declining new reported deaths for a given day, we can draw inference on how many individuals actually died that day. If we assume that the reporting structure is constant over time, we quickly get good estimates of the true number of deaths.

A formal characterization of the model is available in the Appendix A. Here, we only include a simple explanation of how the model operates. Suppose, for example, that on day 1, 4 individuals are reported dead for that day. On the second day, 10 deaths are recorded for day 2. Then, with no further information, it is reasonable to assume that more people died on day 2.

A simplified version of the model could be described as follows: assume that the delay structure is such that 10% of the deaths for a given day are reported on the first day and 5% are reported on the second day. Then, if the first day report is of 20 deaths and the second day report is also of 20 death, assuming each report is drawn from a binomial distribution (calculating the likely number of trials needed for 20 successes with a success probability of 10% and 5% respectively, and where the second draw uses 20 trials less), using Bayes theorem gives a 95% credible interval that the true number of deaths is within the range [207,368] with a mode of 275.

If, in the example above, 60 deaths are reported during the second day to have happened during day 1, and on the third day, only 40 are reported for day 2, we now have conflicting information. From the first-day reports, it seemed like more people had died during day 2, but the second day-reports gave the opposite indication. The model we propose systematically deals with such data and handles many other sources of systematic variation in reporting delay. In fact, the Swedish reporting lag follows a calendar pattern. The number of events reported during weekends is much smaller. To account for this, we allow the estimated proportions of daily reported cases to follow a probability distribution taking into consideration what type of day it is.

Since the pre-print version of this study was published, an article on COVID-19 nowcasting for Germany has appeared [14]. The underlying framework is similar to that employed in this paper, but they model reporting delay with a Poisson (or negative binomial) distribution rather than a binomial distribution. In addition, two nowcasting studies published before the pandemic [15,16] use a Poisson (or negative binomial) count as their likelihood. If the true number of deaths on a given day is small, the Poisson approximation of the binomial count will be wrong (given unbounded support). However, for a moderate to large number of deaths, the Poisson approximation is reasonable, given the law of rare events. To model the underlying pandemic, [14,15] used Brownian motion and [16] used splines, while we are using Gaussian processes with Matérn covariance (of which Brownian motion is a special case). The effect of this on nowcasting accuracy depends on how informative the data are; if a large proportion of cases are being reported in the first days (the likelihood is very informative), then all methods should perform similarly. On the other hand, if only a small part of cases are being reported early, the smoothness of the underlying processes will have a larger effect on the prediction [17]. The estimated function of the latent processes is differentiable approximately once for Sweden and twice for the UK, indicating that the latent epidemic is smoother than Brownian motion.

## 3. Applying the Model to COVID-19 in Sweden and the UK

In this section, we use the model to nowcast daily COVID-19 deaths in Sweden and the UK. We use data published by the Swedish Public Health Agency and by the UK Government. The Swedish data are published in daily snapshots. An archive of these snapshots is required to calculate reporting delays, and is included in the Supplementary Materials. For the UK, the coronavirus data API allows access to historical reports [18]. We calculate the posterior distribution, prediction median and 95% prediction intervals of the expected deaths from the reported deaths on each specific day. The method and algorithm is thoroughly described in the Appendix A. In addition, Appendix B includes the description of a more computationally efficient algorithm that employs a Laplace-like approximation of the full model. During the pandemic, this approximation was used in a nowcast of Swedish death rates that was published online every day [19].

To get accurate estimates, we apply two institution-specific corrections. First, we only count workdays as constituting reporting delay, as very few deaths are reported during weekends. Second, we apply a constant bias correction to account for the fact that Swedish deaths come from two distinct populations with different reporting delay trends: deaths in hospitals and deaths in elderly care.

**Figure 2:**
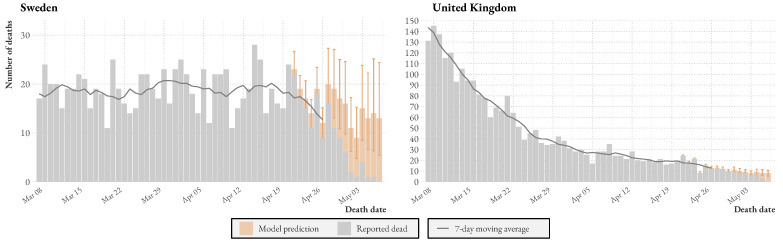
Reported Swedish and UK COVID-19 deaths as of 6 May 2021 and model predictions.

Figure 2 shows a suggestion of how the model could be used to aid decision-making. We apply the model to the latest statistics from Sweden and the UK. The graph shows reported and predicted deaths (with uncertainty intervals) as bars, and a dashed line plots the 7-day (centered) moving average. A version of the plot for Sweden but without predictions was used in the Public Health Agencyś daily press briefings during the first wave. As expected, the model provides estimates of actual deaths considerably above the reported number of deaths for the latest dates. Note that the model predicts additional deaths above the moving average line.

When comparing the two countries, we see that in Sweden, on average, 7% of deaths are reported in the first two days of reporting, while 15% of the remaining cases are reported in the following two days. In contrast, in the UK, 25% of cases are reported in the first two days of reporting, and 50% of the remaining cases are reported in the following days. This suggests that the UK is better equipped to detect sudden increases in deaths and respond promptly based on this information.

### Model Performance

To judge whether the model is accurate, we need to compare it to a benchmark. The moving average of reported deaths is not useful, since it is biased for deaths that occurred within the last week. Instead, we create a benchmark prediction by a normal distribution where the mean and standard deviation are taken from the historical lags from the last two weeks of reported numbers. For a death date 2 days ago, we add the mean of deaths reported after 3 days, 4 days, etc. We use the sum of standard deviations to generate the prediction intervals, assuming that lags are independent across days. The exact calculation is described in the Appendix A.

**Figure 3:**
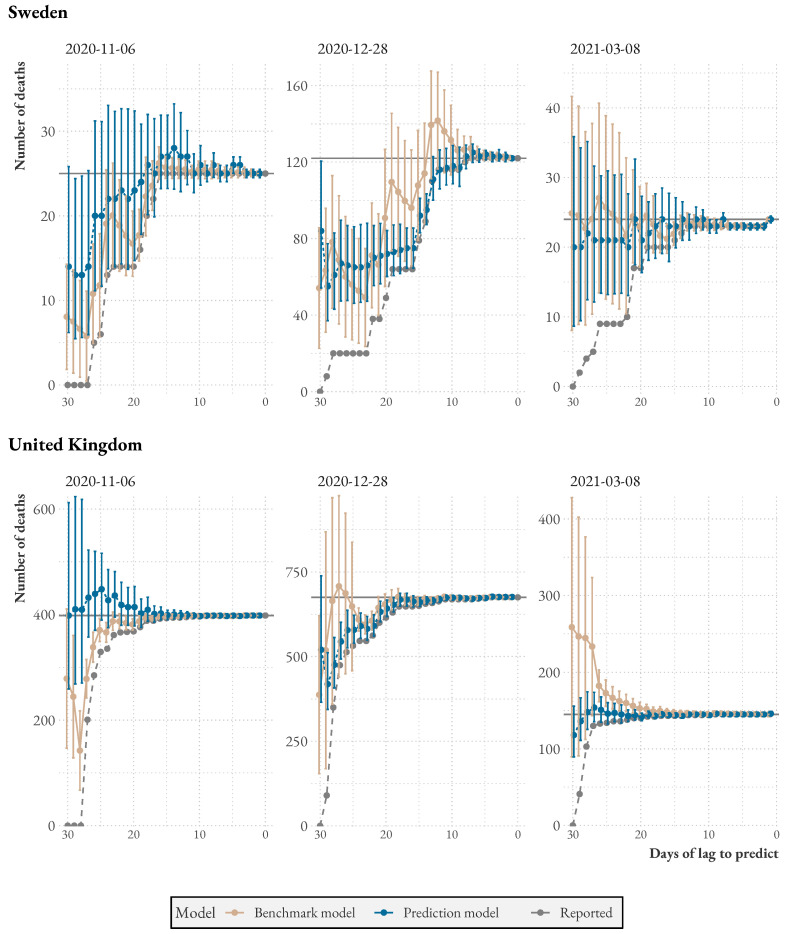
Model accuracy over time for three randomly chosen dates, compared to the constant benchmark. The gray dots indicate the actual number of reported dead until that point in time. The solid line indicates the total number that will have been reported after 30 days.

Figure 3 depicts three randomly chosen dates for Sweden and the UK, respectively, where the model is compared to the benchmark. Both are tasked with predicting the total number of individuals who have died on the given dates and have been reported within 30 days of that date. As time progresses, more deaths are reported, and the dashed gray line approaches the horizontal line. Meanwhile, model uncertainty decreases. Swedish data suffer from considerably longer reporting delays and do not converge until the end of the 30 days, while British statistics converge faster.

**Figure 4:**
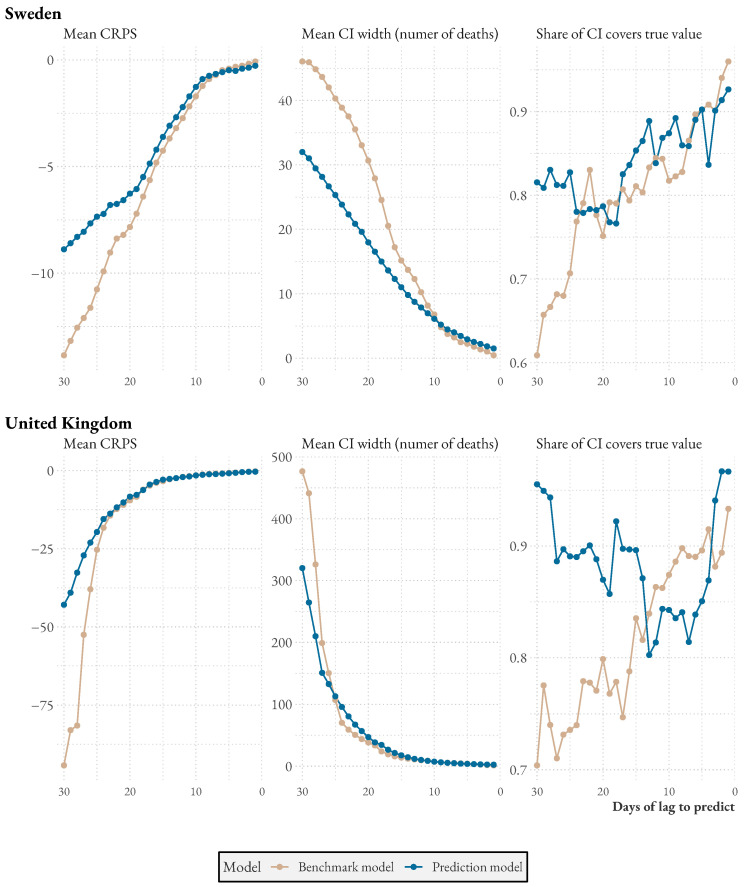
Average (January 2020 to May 2021) model and benchmark accuracy as more information becomes available.

Figure 4 shows model performance compared to the benchmark for three difference performance metrics. All graphs are based on predictions of reported deaths within 30 days and show how performance increases as more data are reported. Each data point is the average of all dates where predictions can be evaluated. CRPS is a measure of accuracy that rewards precision; it is a proper scoring rule, like the continuous probability rank score or the Brier score [20]. The central plot shows the width of the prediction intervals, and the rightmost one the proportion of prediction intervals that cover the true value.

With only one observation (and 29 reporting days left), the CRPS difference for Sweden is 4.93. The difference decreases with more data. With 18 reporting days left or less, the average CRPS difference is below 1. For the UK, the difference starts at 51.79 but decreases quickly, reaching a difference below 1 already with 23 days left. In other words, it is during the first days, when the least amount of data is available and prediction the hardest, that the model really performs well. During this period, the model predictions more often cover the truth while simultaneously reporting tighter uncertainty intervals.

## 4. Discussion

The model proposed here can estimate trends in surveillance data with reporting delays, such as the daily COVID-19 reports in Sweden or the UK. To generate accurate estimates of the actual event frequencies based on these reports is highly relevant and can have large implications for interpretations of trends and the evolution of disease outbreaks. In both countries, delays in reporting increase during the holidays and need to be accounted for to generate valid predictions for these periods. The method and algorithm proposed overcomes major shortcomings in the daily, real-time interpretation of COVID-19 statistics. It also provides valuable measures of uncertainty around these estimates, showing users how large the range of possible outcomes can be.

Whenever case statistics are collected from multiple sources and attributed to its actual event date in the middle of a public health emergency, similar reporting delays to those discussed here will necessarily occur. The method described thus has implications and value beyond the two cases reported, and can be used in any situation where nowcasts of disease event frequencies are of relevance to public health. As we build a stronger pandemic defense for the future, real-time performance indicators will likely play a crucial role. Taking measurement issues explicitly into account will provide significant advantages and help to coordinate the beliefs of decision makers towards the true state.

An interesting avenue could be to use the model together with a model estimating the transmission of a pandemic pathogen to see how reporting delays could impact the understanding of various scenarios, varying the strength of the pandemic the type of reporting delay. For example, if a pathogen starts to spread right before a holiday season, the increasing reporting delay could impact assessments.

Nevertheless, the method also has some limitations. As presented, the model assumes that all deaths are reported in the same manner. For Sweden, disease prevention and control is coordinated at the regional level, and institutional reporting differences likely vary systematically between regions. For example, it is easy to see that the Swedish region Västra Götaland follows a different reporting structure than Stockholm. Building a model for each region separately would most likely give better results and make the assumptions more reasonable. Unfortunately, we do not currently have access to the high-resolution data required to do so.

Moreover, deaths are reported from two distinct populations that seem to follow different trends. When the first version of this paper was written, daily deaths in elderly care were reported with a longer delay and seemed to be decreasing more slowly than hospital deaths. However, statistics offer only aggregate numbers, prohibiting us from modeling these two distinct processes separately.

Other systematic patterns can be addressed with the aggregate data we have used. We noted a clear decline in proportions of deaths reported during the two first working days of each week in Sweden. For example, for 2 April 2020, ≈30% of deaths were reported within the first two working days, whereas for 18 May 2020, only ≈10% were reported during this period. A likely explanation is that reporting routines were adapted to normal work schedules. Such systematic changes in reporting delay will hurt model accuracy if not explicitly modeled. Since the model places more weight on recent information, the negative impact of one-off institutional changes decreases with time, however. Continuous changes in reporting delay that are not modeled will induce persistent bias and decrease performance. This is true for any prediction model, as well as the benchmark used here. By explicitly modeling the variation in reporting delay, the performance advantage over the benchmark model would only increase.

## 5. Conclusions

In this paper, we provide a method to accurately nowcast daily COVID-19 statistics that are reported with delay. By systematically modeling the delay, policymakers can avoid dangerously illusory downward trends. Our model also gives precise uncertainty intervals, making sure that users of these statistics are aware of the fast-paced changes that are possible during a pandemic. By improving the accuracy and speed of data reporting, our proposed methodology helps to alleviate one of the key problems underscored by the Lancet commission on COVID-19 [2].

## Figures and Tables

**Figure A1:**
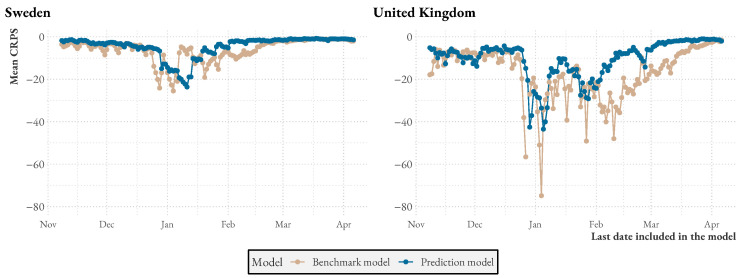
Mean CRPS as the pandemic progresses.

**Figure A2:**
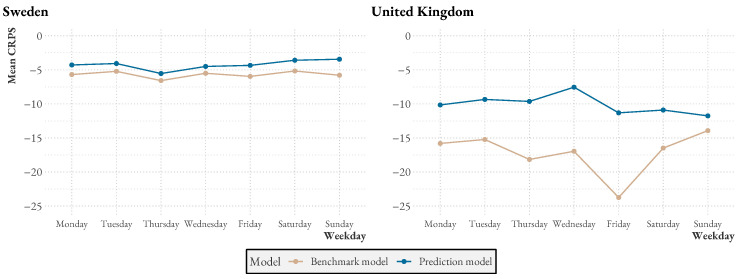
Mean CRPS per weekday.

## Data Availability

Data and code for this project is available at https://github.com/adamaltmejd/covid_reporting_delay_prediction (accessed on 1 November 2022).

## Supplementary Materials

The following supporting information can be downloaded at: https://github.com/adamaltmejd/covid_reporting_delay_prediction (accessed on 1 November 2022).

## Appendix A. Model

We use a Bayesian version of the removal model that assumes an over-dispersed binomial distribution for the daily observations of COVID-19 deaths in Sweden. The reason for using a Bayesian approach is that it is relativity easy to incorporate external information. It allows us, for instance, to include a latent Gaussian process to model the underlying pandemic.

### Appendix A.1. Notation

Before presenting the model, we describe some notation used throughout the appendix. For an m×n matrix, *r* we use the following broadcasting notation: $r_{k,j:l}=\left[r_{k,j},r_{k,j+1},\ldots ,r_{k,l}\right]$.

Further, x|y∼π(.) implies that the random variable *x*, if we condition on *y*, follows the distribution π(.).

The relevant variables in the model are the following:

**Variable Name**	**Dimension**	**Description**
d	T×1	$d_{i}$ is the number of deaths that occurred on the day *i*.
r	T×T	$r_{ij}$ is number of death recorded for day *i* at day *j*. Note that $r_{ij}$ for i<j is not defined.
p	T×T	$p_{ij}$ is the probability of that a death for day *i* not yet recorded is recorded at day *j*. Note that $p_{ij}$ for i<j is not defined.
α	K×1	latent prior parameter for p
β	K×1	latent prior parameter for p
$\alpha^{H}$	2×1	parameter for the probability, p for holiday adjustment
$\beta^{H}$	2×1	parameter for the probability, p for holiday adjustment
μ	T×1	$\mu_{i}$ is the intensity of the expected number of deaths at day *i*
$\sigma^{2}$	1×1	variation of the random walk prior of the log intensity
ϕ	1×1	overdispersion parameter for the negative binomial distribution
$p_{0}$	1×1	probability of reporting for a low reporting event
π	1×1	probability of a low reporting event

### Appendix A.2. Likelihood

The most complex part of our model is the likelihood, i.e., the density of the observations given the parameters. Here, the data consist of the daily report of recorded deaths for the past days. This can conveniently be represented by an upper triangular matrix, r, where $r_{i,j}$ represents the number of new reported deaths for day *i* reported at day *j*. This matrix is displayed in Table A1.

**Table A1:** The matrix describes how the data are structured.

	Reported Date				
**Death Date**	$r_{11}$	$r_{12}$	⋯	⋯	$r_{1T}$
		$r_{22}$	⋯	⋯	$r_{2T}$
			$r_{33}$	⋯	$r_{3T}$
				⋱	⋮
					$r_{TT}$

We assume that given the true number of deaths on day *i*, $d_{i}$, that for each reported day *j*, the remaining deaths $d_{i}-\sum_{k=1}^{j-1}r_{i,k}$ are each recorded with probability $p_{ij}$, i.e.,

$$r_{i,j}|d_{i},r_{1,1:j}.p∼Bin\left(d_{i}-\sum_{k=1}^{j-1}r_{i,k},p_{i,j}\right).$$

Typically, in removal sampling, one would set the probability of reporting uniformly, i.e., $p_{i,j}:$ = *p*. However, for these data, this is clearly not realistic given weekly patterns in reporting—there is very little reporting during the weekends. Instead, we assume that we have *k* different probabilities. Further, to account for over-dispersion, we assume that each probability, rather being a fixed scalar, is a random variable with a Beta distribution. The Beta distribution has two parameters: α and β. This results in the following distribution for the probabilities:

$$p_{i,j}|\alpha ,\beta ,\alpha^{H},\beta^{H}∼Beta\left(\alpha_{j}^{H}\alpha_{min(j-i,k)},\beta_{j}^{H}\beta_{min(j-i,k)}\right).$$

Here, if j∈H, then day *j* is a holiday or weekend, and the parameters above are

$$\alpha_{j}^{H}=\left\{\begin{array}{cc} \alpha_{1}^{H}\alpha_{2}^{H} & if\{j\in H\}\cup \{j-1\in H\},\\ \alpha_{1}^{H} & if\left\{j\in H\right\}\cup \left\{j-1\in H^{c}\right\},\\ \alpha_{2}^{H} & if\left\{j\in H^{c}\right\}\cup \left\{j-1\in H\right\},\\ 1 & else, \end{array}\right.$$

and

$$\beta_{j}^{H}=\left\{\begin{array}{cc} \beta_{1}^{H}\beta_{2}^{H} & if\{j\in H\}\cup \{j-1\in H\},\\ \beta_{1}^{H} & if\left\{j\in H\right\}\cup \left\{j-1\in H^{c}\right\},\\ \beta_{2}^{H} & if\left\{j\in H^{c}\right\}\cup \left\{j-1\in H\right\},\\ 1 & else. \end{array}\right.$$

These extra parameters are created to account for the under-reporting that occurs during weekend and holidays.

Finally, we add an extra mixture component that allows for very low reporting.

### Appendix A.3. Priors

For the α and β parameters, we use an (improper) uniform prior. For deaths, d, one could imagine several priors, ideally based on some sort of epidemiological model. However, here, we simply assume a log-Gaussian Cox process [21]. Instead of a Poisson distribution, we use a negative binomial to handle possible over-dispersion. The latent Gaussian processes is defined by its covariance function. We use a Whittle-Matern covariance function. This results in the following mode:

$$\begin{array}{cc} \log(\mu ) & ∼N(0,\Sigma ),\\ d_{i}|\mu_{i} & ∼NegBin(\mu_{i},\phi ). \end{array}$$

This model is created to create a temporal smoothing between the reported deaths. For the hyperparameter $\sigma^{2}$, we impose an inverse Gamma distribution; this prior is suitable here because it guarantees that the process is not constant ($\sigma^{2}=0$), which we know is not the case.

### Appendix A.4. Full Model

Putting the likelihood and priors together, we get the following hierarchical Bayesian model:

$$\begin{array}{cc} \sigma^{2} & ∼\Gamma (1,0.01)\\ \phi & ∼\Gamma (1,0.01)\\ \alpha_{k} & ∼U[0,\infty ]\\ \beta_{k} & ∼U[0,\infty ]\\ \alpha_{k}^{H} & ∼U[0,\infty ]\\ \beta_{k}^{H} & ∼U[0,\infty ]\\ \log\left(\mu_{i}\right)-\log\left(\mu i-1\right) & ∼N(0,\sigma^{2})\\ d_{i}|\mu_{i} & ∼NegBin(\mu_{i},\phi )\\ p_{i,j}|\alpha ,\beta ,\alpha^{H},\beta^{H} & ∼Beta(\alpha_{j}^{H}\alpha_{min(j-i,k)},\beta_{j}^{H}\beta_{min(j-i,k)})\\ r_{i,j}|d_{i},r_{1,1:j},p & ∼\pi Bin\left(d_{i}-\sum_{k=1}^{j-1}r_{i,k},p_{0}\right)+\left(1-\pi \right)Bin\left(d_{i}-\sum_{k=1}^{j-1}r_{i,k},p_{i,j}\right), \end{array}$$

where and j≤i and i=1,…,T.

## Appendix B. Inference

We generate inference about the number of deaths through the posterior distribution of d given the observations r. To sample from this distribution, we use the Markov Chain Monte Carlo method [22]. We employ a blocked Gibbs sampler, which generates samples in the following sequence:

- First, we sample $\alpha ,\beta ,\alpha^{H},\beta^{H}|d,r$. Using the fact that one can integrate out *p* in the model, $d|\alpha ,\beta ,\alpha^{H},\beta^{H},r,\lambda$ follows a Beta-Binomial distribution. Here, we also use an adaptive MALA [23] to sample from these parameters.
- To sample $d|\alpha ,\beta ,\alpha^{H},\beta^{H},r,\lambda$, we assume that each death, $d_{i}$ is conditionally independent, and use a Metropolis Hastings random walk.
- To sample $\lambda |d,\sigma^{2}$, we again use an adaptive MALA.
- Finally, we sample $\sigma^{2}|d$,and $p_{0},\pi$ directly, since this distribution is explicit, and ϕ using an MH-RW.

### Simplified Model

In order to make the model run quickly and update daily, we present a simplified version of the model. Running the full MCMC algorithm each day is both slow and sometimes leads to poor mixing (this effect is due to the latent Gaussian processes, defined by equation $\log\left(\mu_{i}\right)-\log\left(\mu i-1\right)∼N\left(0,\sigma^{2}\right)$). Here, due to the fact that $d_{i}$ is a discrete unknown, we cannot use RStan [24], which would have been ideal. Instead, we make a Box–Cox approximation of the latent processes:

$$\begin{array}{c} \sqrt{d}∼N\left(\mu ,\Sigma \right), \end{array}$$

where we assume the square root of the number of deaths follows a Matérn process [25]. However, as we do not observe the number of deaths, we also make a normal approximation of the capture removal likelihood, and the approximated model is thus

$$\begin{array}{cc} \sqrt{d} & ∼N\left(\mu ,\Sigma \right),\\ {\sqrt{d}}_{i}|r_{i,i:j} & ∼N\left({\hat{\mu }}_{ij},{\hat{\sigma }}_{ij}^{2}\right). \end{array}$$

Since the approximate distribution of $\sqrt{d}$ is a Gaussian process, prediction and confidence bounds are explicit. In more detail:

- We fit α,β for the retain sampling, treating $d_{i}$ as known after 30 days, using a MAP estimate.
- We then run an MCMC chain on the likelihood part of $d_{i}$ for the fixed parameters in the previous step;
  $$d_{i}|r_{i,i:j}\propto BB\left(r_{i,i:j},d_{i}\alpha ,\beta \right).$$
  This MCMC has no mixing problem as each $d_{i}$ is independent of each other and one can adapt each chain separately. Note that this is the model from the previous section but with no prior on $d_{i}$, i.e., no log-Gaussian Cox processes.
- Since the number of deaths approximately follows a Poisson distribution, we make a Box–Cox transformation and assume that the square root of the number of deaths is approximately normal:
  $$\sqrt{d}∼N\left(\mu ,\Sigma \right).$$
  To link the catch retain model to the Box–Cox model, we make a normal approximation of the posterior distribution. We approximate the posterior distribution with
  $${\sqrt{d}}_{i}|r_{i,i:j}\approx N\left({\hat{\mu }}_{ij},{\hat{\sigma }}_{ij}^{2}\right).$$
  where ${\hat{\mu }}_{ij}$ and ${\hat{\sigma }}_{ij}^{2}$ are the MCMC estimate of the posterior mean and variance obtained from above. This is roughly a Monte Carlo Laplace approximation. The latent parameters $\left(\mu ,\Sigma \right)$ are fitted using maximum likelihood.
- Using the above model, we get an explicit posterior distribution of the square root of the number of deaths. In order to generate predictions of the number of deaths, we simulate the square number of deaths, setting negative values to zero.

## Appendix C. Model Benchmark

In this section, we describe the benchmark model in detail and present additional comparisons of the model to the benchmark.

The benchmark model simply takes the sum of average historical reporting lags for the preceding 14 days. As before, $r_{ij}$ is the number of deaths that happened on day *i* and were recorded on day *j*. To predict the number of people who died on a given day, we first calculate lag averages:

(A1) $$\begin{array}{c} {\hat{r}}_{i,i+L}=\frac{\sum_{k=i-14}^{14}r_{k-L,k}}{14}, \end{array}$$

where ${\hat{r}}_{i,i+L}$ is the average number of deaths reported with a lag of *L* days, based on the 14 reports closest preceding day *i*. If we are looking at data released 28 April 2020 and call this day 0, the latest death date that we have 10-day (L=10) reporting lag observation for is $r_{-10,0}$. The average for Lag(0,10) is therefore taken over the 14 days between $r_{-24,-14}$ and $r_{-10,0}$ (4 April 2020 and 18 April 2020). The average is then taken over all available reports.

In the comparisons, we aim at predicting the total number of deaths that will have been reported within 14 days of the death date. To do so, we sum over the average lag that has yet to be reported. If we are predicting the number of people who have yet to be reported dead for day −3, we already know the true values for $r_{-3,-3}$, $r_{-3,-2}$, $r_{-3,-1}$, and $r_{-3,0}$, so we only need to predict $r_{-3,1}\ldots r_{-3,10}$. The prediction is then

(A2) $$\begin{array}{c} Benchmark\left(i,j\right)=\sum_{l=i}^{j}r_{i,l}+\sum_{l=j}^{14}{\hat{r}}_{i,l}. \end{array}$$

To calculate confidence intervals, we simply use a normal assumption with standard deviations of the reporting lags, assuming independence, i.e., this is just the square root of the sum of $Var(\hat{r})$.

Figure A1 presents additional statistics comparing the model to the benchmark over time and over the week. We see that both model and benchmark performances drop during similar periods, but that also, during these times, the model performance is usually higher. Over the week, especially in the UK, the benchmark seems to have some trouble with Fridays, likely because weekend reporting drops have not been explicitly modeled.
